# Acquisition Duration in Resting-State Arterial Spin Labeling. How Long Is Enough?

**DOI:** 10.3389/fnins.2020.00598

**Published:** 2020-07-30

**Authors:** Corentin Vallée, Pierre Maurel, Isabelle Corouge, Christian Barillot

**Affiliations:** Université de Rennes, Inria, CNRS, Inserm, IRISA UMR 6074, Empenn ERL U-1228, Rennes, France

**Keywords:** functional magnetic resonance imaging, arterial spin labeling, resting-state fMRI, acquisition duration, modeling

## Abstract

Resting-state Arterial Spin Labeling (rs-ASL) is a rather confidential method compared to resting-state BOLD. As ASL allows to quantify the cerebral blood flow, unlike BOLD, rs-ASL can lead to significant clinical subject-scaled applications. Despite directly impacting clinical practicability and functional networks estimation, there is no standard for rs-ASL regarding the acquisition duration. Our work here focuses on assessing the feasibility of ASL as an rs-fMRI method and on studying the effect of the acquisition duration on the estimation of functional networks. To this end, we acquired a long 24 min 30 s rs-ASL sequence and investigated how estimations of six typical functional brain networks evolved with respect to the acquisition duration. Our results show that, after a certain acquisition duration, the estimations of all functional networks reach their best and are stabilized. Since, for clinical application, the acquisition duration should be the shortest possible, we suggest an acquisition duration of 14 min, i.e., 240 volumes with our sequence parameters, as it covers the functional networks estimation stabilization.

## 1. Introduction

Functional MR imaging (fMRI) builds the links between location and function in the brain. The two main sub-domains in fMRI are task-based fMRI and resting-state fMRI. In task-based fMRI, the location of a function is considered to be where the acquired signal matches with the task guidelines given to the subject (Poldrack et al., 2011). In resting-state fMRI (rs-fMRI), as no task is given, the focus is on fluctuations in voxels time-series induced by spontaneous neural activations. Similarities in these time-series in different areas have been shown to not be random but to be matching the function of the brain (Biswal et al., 1995). These similarities between neural activation pattern define the *functional connectivity* of the brain, and show the underlying cerebral architecture is organized into functional specialized units, called networks, communicating with each other (Varoquaux and Craddock, 2013; Fan et al., 2016). Resting-state functional imaging aims to identify functional networks of the brain and to depict how they interact outside any structural connectivity consideration (Van den Heuvel and Hulshoff Pol, 2010). As the resting-state is rather easy to achieve, especially for the pediatric and elderly population but also for cognitive impaired patients, rs-fMRI has found some niche clinical applications in diseases investigation. The key concept is to consider the brain as a unique integrative network of the estimated functional networks, assimilated with the mathematical structure of a graph (Van den Heuvel and Hulshoff Pol, 2010; Wang et al., 2010). The graph properties model the functional architecture: each node corresponds to a functional network, and edges configuration depicts how they interdepend. For instance, functional connectivity is altered in disease like schizophrenia (Lynall et al., 2010; Kindler et al., 2015), major depression (Mulders et al., 2015), Alzheimer's disease (Sanz-Arigita et al., 2010; Agosta et al., 2012). Rs-fMRI, as a global investigation of function, is also appropriate for neurodegenerative diseases like multiple sclerosis (Faivre et al., 2012; Filippi et al., 2013; Cruz-Gómez et al., 2014), Parkinson's disease (Gao and Wu, 2016), or amyotrophic lateral sclerosis (Mohammadi et al., 2009; Trojsi et al., 2017).

Another subdivision in fMRI concerns the way the signal is obtained. The two major techniques are Blood Oxygenation Level Dependent (BOLD) fMRI and functional Arterial Spin Labeling (fASL). Based on neurovascular coupling effects, BOLD techniques rely on the local signal variation induced by the neuron consumption of blood oxygen (Kim and Ugurbil, 1997). Arterial Spin Labeling (ASL) is an MRI perfusion technique that uses magnetically labeled arterial water protons as an endogenous tracer (Detre et al., 1992). An inversion pulse labels the inflowing blood, and, after a delay called the post-labeling delay, a labeled image of the volume of interest is acquired. The subtraction of the labeled image from a control image, i.e., non-labeled, reflects the quantity of spins that have perfused the imaged volume, producing what is commonly called a perfusion-weighted (PW) image. The PW map can be used to quantify the cerebral blood flow (CBF) under some assumptions (Buxton et al., 1998; Borogovac and Asllani, 2012). The absence of a contrast agent injection makes ASL well-suited for CBF longitudinal studies, particularly for a pediatric population or for a population with poor venous access or contrast agent contraindication. The quantification of CBF is the main advantage of ASL over BOLD. Indeed, a BOLD signal provides an indirect and non-quantitative measurement of neural activity, as it results from a combination of variations in CBF, cerebral blood volume, and cerebral metabolic rate of oxygen (Buxton, 2010). The main drawback of ASL is its lower signal-to-noise ratio compared to BOLD fMRI. The repetition time (TR) is also twice to three times higher in rs-ASL compared to resting-state BOLD (rs-BOLD), which impacts its temporal resolution. Furthermore, ASL can be implemented through numerous MRI sequences, and meta-analyses can be difficult to set up, as ASL shows a high sequence parameter dependency (Buxton et al., 1998; Grade et al., 2015; Mutsaerts et al., 2015). Nevertheless, a consensus seems to be emerging over the years (Alsop et al., 2015). BOLD is, however, predominant in clinical usage and in academic research and is therefore considered the gold standard in rs-fMRI. Consequently, rs-ASL has been mostly used for research purposes. However, the recent increasing involvement of all MRI stakeholders is highly beneficial in order to further develop rs-ASL usage in terms of a guide for clinical practitioners (Grade et al., 2015) as well as consensus on implementation (Alsop et al., 2015), feasibility, and viability (Chen et al., 2015). Despite its lower SNR, but thanks to its closer proximity to neural activation (Duong et al., 2001; Tjandra et al., 2005) and CBF quantification, rs-ASL has been proven to be a serious contender to resting-state BOLD in schizophrenia (Zhu et al., 2015) and Alzheimer's disease (Alsop et al., 2010; Zhang et al., 2017). Resting-state ASL has also found its own clinical applications with investigation of chronic fatigue syndrome (Boissoneault et al., 2016) and catatonia (Walther et al., 2016).

The acquisition duration is an important parameter in an rs-fMRI study with strong practical consequences. Most current rs-ASL studies work with a duration from 8 to 13 min and a TR from 3 to 4 s, i.e., 120–260 images. Intuitively, one would assume the longer the duration the better the sampling of the signal correlation across the brain and, thus, the better the acquisition. However, this requires us to define what “better” actually means and does not consider the practical questions of clinical implementation and subject resting-state upholding. To the best of our knowledge, some papers already studied the influence of duration in rs-BOLD (Van Dijk et al., 2010; Anderson et al., 2011; Birn et al., 2013; Laumann et al., 2015; Termenon et al., 2016; Bouix et al., 2017), whereas, in rs-ASL, it has not been explored yet. In this work, we first focus on the feasibility of detecting functional networks with rs-ASL. We remain as close as what a typical investigator of rs-ASL would experience by implementing usual sequence, processing, and functional networks detection methods. We then assess a trend over the duration influence on rs-ASL detected networks quality: we do not directly assess whether an acquisition is good at a given time but rather how it evolves with longer durations. After describing the scores used and the modeling approach, an in-depth analysis for the Default-Mode Network (DMN) will be presented in order to illustrate scores evolution on the most typical resting-state network. Finally, we will show results for all the functional networks under consideration and discuss a recommended sequence duration in rs-ASL.

## 2. Materials

### 2.1. Subjects

Seven healthy male right-handed subjects aged from 21 to 28 years (23.5 yo ± 2.5) were involved in this study. All subjects gave written informed consent before participating in the study. We have maintained the homogeneity of the population in order to limit the influence of factors, such as gender or age.

### 2.2. MR Acquisitions

The subjects were scanned on a 3.0T whole body Siemens MR scanner (Magnetom Verio, Siemens Healthcare, Erlangen, Germany) with a 32-channel head coil. A 3D anatomical T1-weighted MP2RAGE image was acquired for each subject. The resting-state ASL imaging was performed using a 2D EPI pseudo-continuous (pCASL) sequence. Subjects were asked to keep their eyes closed, to relax (mind-wandering), to lie still and to not fall asleep. We used common parameters reported in the literature: TR = 3,500 ms, FoV = 224 × 224 mm^2^, TE = 12 ms, LD = 1,500 ms and a 1250 ms post-labeling delay (PLD) at the first slice (1712.5 ms at the median slice). Volumes were made of 24 slices of 64 × 64 voxels with 5 mm slice thickness with 20% gap for a total resolution of 3.5 × 3.5 × 6 mm^3^. The number of volumes was 420 for a total duration of 24 min 30 s.

### 2.3. Data Pre-processing

**Figure 1:**
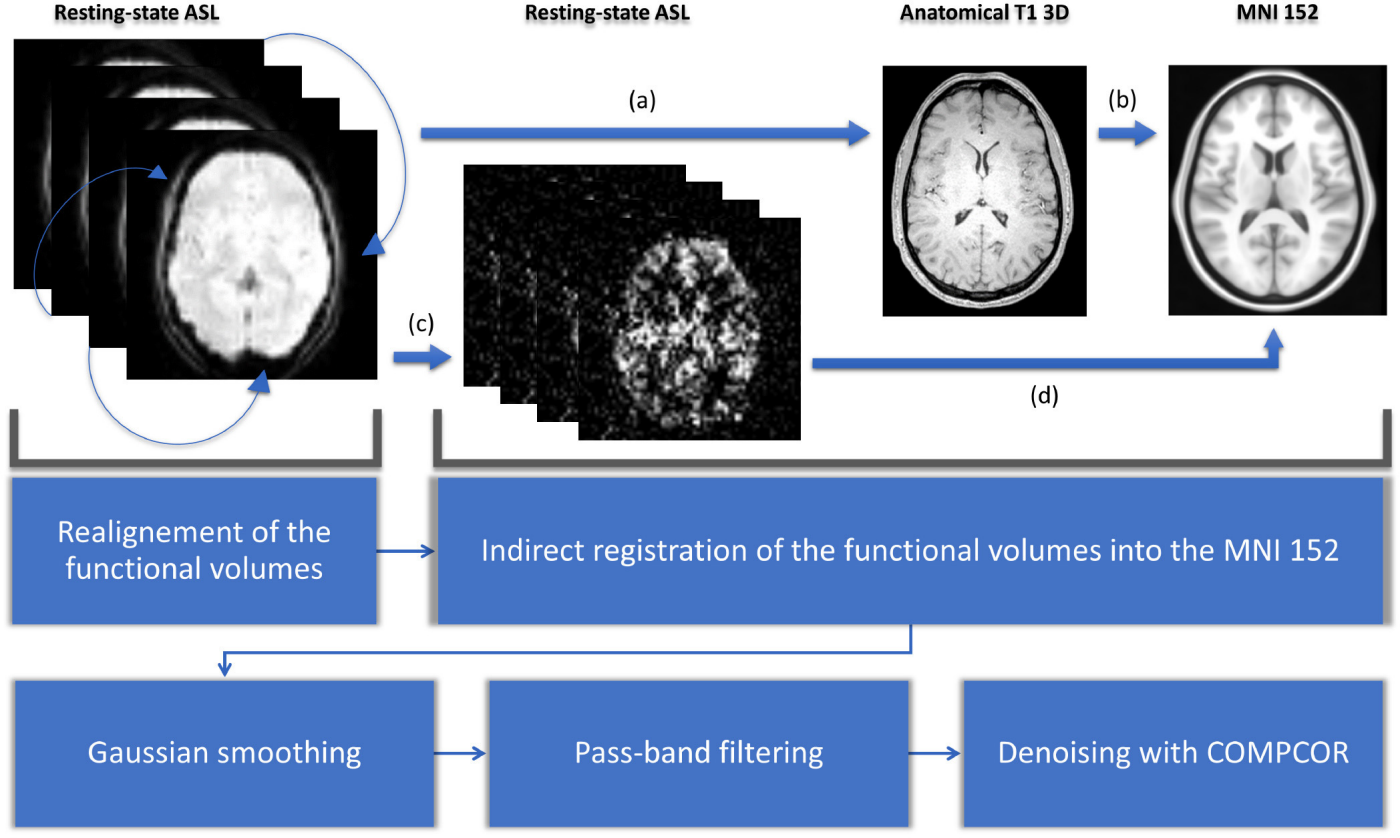
Preprocessing starts with a realignment: all the functional volumes are registered with the first one. Second step is the indirect normalization of the functional volumes. The functional data is registered on the anatomical 3D T1 (a). Then the anatomical image is registered on the MNI 152 template (b). After a surround subtraction (c), transformations of (a) and (b) are composed to register functional data on the MNI template (d). We then used Gaussian smoothing with a typical 6 mm FWHM radius and pass-band filtering with a range from 0.005 to 0.1 Hz. Final denoising is made with COMPCOR.

For each subject, the raw pCASL series is divided into 46 sub-series. The duration of these sub-series ranges from nearly 2 min (34 volumes) to 24 min 30 s (420 volumes) with a time step of 30 s. For the sake of simplicity, we will only mention rounded durations hereafter. All these subdivisions are made *before* any preprocessing: the preprocessing is done independently on each sub-series. For the preprocessing steps and their parameters, we chose the most common ones found in bibliography. While identifying the best preprocessing is still an open topic in rs-ASL in particular and in rs-fMRI in general (Gargouri et al., 2018), a general routine can be discerned in the literature. For the preprocessing steps, we used Matlab CONN toolbox (www.nitrc.org/projects/conn, RRID:SCR_009550) (Whitfield-Gabrieli and Nieto-Castanon, 2012). Preprocessing starts with the realignment of the functional data. All the functional volumes are rigidly registered to the first one using SPM12 realign procedure (Andersson et al., 2001). Second step is the indirect normalization of the functional volumes (Nieto-Castanon, 2020). It starts with the affine registration of the functional data, with the mean volume of the realigned series as the source image, on the anatomical 3D T1 using SPM12 inter-modality co-registration procedure with a normalized mutual information cost function. Then the anatomical image is registered on the MNI 152 template using SPM12 unified segmentation and normalization procedure (Ashburner and Friston, 2005). In parallel, perfusion-weighted maps are computed using a surround subtraction on the realigned functional data. Both transformations (functional to structural, structural to MNI) are composed to register subtracted functional data on the MNI template. We then used Gaussian smoothing with a typical 6 mm FWHM radius (Alakörkkö et al., 2017) and pass-band filtering with a range from 0.005 to 0.1 Hz. Final denoising was made with aCOMPCOR, using five regressors for white-matter, five for cerebro-spinal fluid, and the 12 from the realignment step (Behzadi et al., 2007). All these steps are shown in Figure 1.

## 3. Methods

### 3.1. Detecting Networks With Seed-Based Analysis

To obtain the mapping of individual functional networks, we relied on seed-based analysis (SBA) (Van den Heuvel and Hulshoff Pol, 2010). The principle of this method, the first proposed to define functional connectivity (Biswal et al., 1995), is quite straightforward. Considering a similarity measure [usually linear correlation, but many others exist (Zhou et al., 2009)], SBA builds functional areas by gathering voxels which exhibit a matching signal, in the sense of the chosen measure, to that of a ROI, called the seed in this context. Even if SBA is a very polymorphic modeling method, we used its most common form in our work. We therefore considered linear correlation as the similarity measure and used a set of 20 single voxels as seeds. Seeds are spread in the expected location of six usual functional networks: DMN, Sensori-motor, Language, Salience, Visual, and Cerebellum. The exact positions of the seeds in the MNI152 space are provided in the Appendix section and were suggested by the CONN toolbox. Each seed provides a linear correlation map of the brain. To estimate a functional network for each seed, we statistically tested whether the signal between the seed and a candidate voxel is positively correlated with a risk of 1% FWER-corrected with Bonferroni procedure. This is a tough conservative testing compared to most of rs-ASL (even fMRI in general) studies, but we agree with the recommendation of Eklund et al. (2016) on false positives underestimation in fMRI literature.

### 3.2. Evaluation Scores

**Figure 2:**
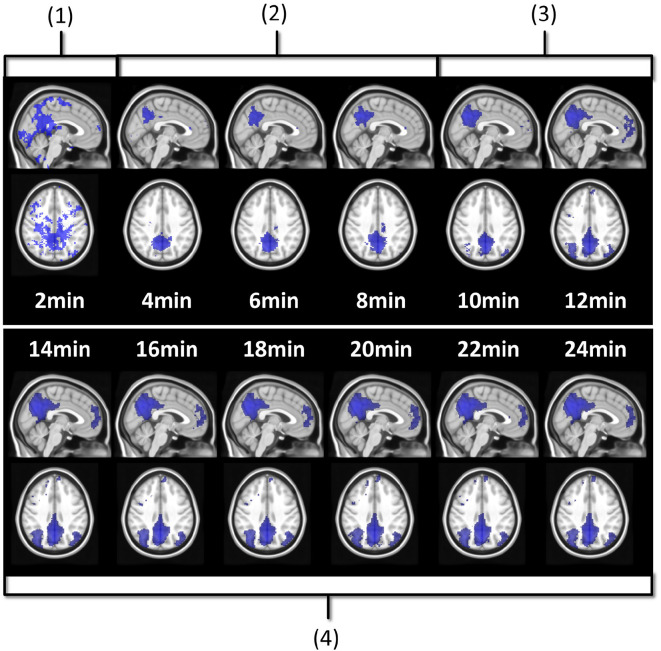
Seed-based estimation of subject 3 DMN with posterior seed at different acquisition durations. Four stages can be identified. At 2 min (1), the map mostly shows false positive noise detection. Between 4 and 8 min (2), the false positive noise has disappeared while the posterior component of the DMN starts growing. Between 10 and 12 min (3), the posterior component is well-detected, and the frontal and the lateral components have appeared, but quite poorly. At 14 min and after (4), DMN detection is good and interestingly, stable.

The resting-state BOLD literature suggests different acquisition durations: 6 min (Van Dijk et al., 2010), 10 min (Bouix et al., 2017), 12 min (Birn et al., 2013), 25 min (Anderson et al., 2011), and even 100 min (Laumann et al., 2015). In Termenon et al. (2016), Termenon et al. focus on the tradeoff that can be made between duration and number of subjects in a group study. They recommend durations ranging from 7 min for 100 subjects to 14 min for 40 subjects. The main reason of their apparent discrepancy is the modeling. Indeed, there are many ways to properly define a model to assess the role of acquisition duration (*a fortiori* how much duration is enough), even if they lead to different conclusions. As a pioneer work on rs-ASL, we wanted our modeling to reflect an investigator's experience with the impact of acquisition duration on functional network estimation. Figure 2 illustrates the investigation of acquisition duration we will model. The DMN estimation is validated after 14 min on Figure 2 because it matches with how the functional network is expected to look like. In modeling terms, it is basically assessing the overlap of the estimated network with a reference network. In order to investigate a trend afterwards and decide which acquisition duration is enough, the individual functional maps were compared to a reference, as in process described in Figure 2. For that purpose, we relied on the Multi-Subjects Dictionary Learning atlas (MSDL) by Varoquaux et al. (2011). The MSDL is an atlas of 17 resting-state functional networks containing our six networks of interest and from which our seeds and subjects are independent. The key idea is to have functional maps close to what an expert would expect to observe when looking for the typical functional areas investigated here. To study the quality of the detected networks as a function of the acquisition duration, we evaluated the overlap between the SBA estimated functional maps and the MSDL references (simply called “reference” hereafter) through two measures: the Jaccard's index and the area under curve (AUC).

Let E be a set, let ${\left(v_{i}\right)}_{i⩽k\in N}$ be observations in E and $\left(M_{1};M_{2}\right)\in {\left\{0;1\right\}}^{E}\times {\left\{0;1\right\}}^{E}$ binary categorical variables. In fMRI, E is the voxels space, the *v*_*i*_ are the voxels, and the variables *M*_1_, *M*_2_ are the functional networks to be compared, which associate to each voxel *v*_*i*_: 1 if the voxel belong to the functional network, 0 otherwise. The definition of *M*_1_ and *M*_2_ is binary here, but it can easily be extended to probability maps. In our case, *M*_1_ corresponds to the estimation of a functional network, and *M*_2_ corresponds to the functional network reference from the MSDL. Let *A*,*B*,*C*,*D* be four sets with the respective cardinals *a*,*b*,*c*,*d* defined by

(1) $$\{\begin{array}{c} A\text{ := }\{v_{i}|M_{1}(v_{i})=1,M_{2}(v_{i})=1\}\\ B\text{ := }\{v_{i}|M_{1}(v_{i})=1,M_{2}(v_{i})=0\}\\ C\text{ := }\{v_{i}|M_{1}(v_{i})=0,M_{2}(v_{i})=1\}\\ D\text{ := }\{v_{i}|M_{1}(v_{i})=0,M_{2}(v_{i})=0\} \end{array}$$

Almost all common similarity measures (Sokal measure family, Sørensen-Dice, correlation etc.) can be defined with *a*,*b*,*c*,*d*, e.g., Sørensen-Dice score: 2*a*/(2*a* + *b* + *c*). As *M*_2_ (MSDL reference) can be considered as a ground-truth, therefore *a* becomes the number of *True Positives*, *b* of *False Positives*, *c* of *False Negatives* and *d* of *True Negatives*. We also have the *Sensitivity*: *a*/(*a* + *c*), *Specificity*: *d*/(*d* + *b*), and the *Positive Predicted Value* (PPV): *a*/(*a* + *b*).

#### 3.2.1. Jaccard's Index

When comparing two spatially distributed data like functional networks, a straightforward measure is the Jaccard's index. It is the ratio between the size of the functional networks' intersection and their union. It is defined by *J* = *a*/(*a* + *b* + *c*) in our notation system. It provides intuitive and visual information about the overlap between one SBA estimation of functional network and one reference network from the MSDL. It is also test-dependent: changing the risk or the multiple comparisons correction at the estimation step will also change the shape and extent of the functional network, generally modifying Jaccard's index. This may be considered as a drawback, but, in fact, a statistical test is usually used at some point when investigating fMRI data.

#### 3.2.2. Receiver Operating Characteristic Analysis

In this section, we assume that the binary categorical variables are parameterized by at least one parameter. For example, in our case, it could be the *p*-value for the statistical test of correlation α that was used to estimate functional networks from the correlation map. Let *r* be our parameter, *a*_*r*_, *b*_*r*_, *c*_*r*_, and *d*_*r*_ the previously defined cardinals in (1) now parameterized by *r*, and let define a set {(*x*(*r*), *y*(*r*)), *r* ∈ [−1, 1]} ⊂ [0, 1]^2^ by

(2) $$\{\begin{array}{l} x(r)=1-\frac{d_{r}}{d_{r}+b_{r}}\\ y(r)=\frac{a_{r}}{a_{r}+c_{r}} \end{array}$$

The implicitly defined function *f* : *x* ↦ *y* is called the *Receiver operating characteristic curve* (ROC-curve), and its integral $\int_{0}^{1}f\left(x\right)dx$ is simply called the *Area Under Curve* (AUC). In the case where *M*_2_ is considered to be the truth, *f* is just informally *f* : 1 − *Specificity* ↦ *Sensitivity*. The AUC has the interesting property to not be test-dependent, as it covers all possible values of the threshold parameter (e.g., risk/correlation). It illustrates how a functional map can be close to the reference by considering all values of the considered parameter, while the Jaccard's index reflects how it is close to the reference by considering one value of the given parameter. Hence AUC seems a better way to assess the trend of interest from a theoretical point of view. However, it is further away from the practical proximity and easier interpretation of the Jaccard's index modeling offers.

### 3.3. Modeling Scores Evolution With Respect to the Duration

With each of the 20 seeds are 322 associated functional network estimations that correspond to seven estimations per each of the 46 different acquisition durations (seven subjects, 46 sub-series). For each seed, and for one of the six functional network references from the MSDL, both Jaccard's index and AUC are computed between the reference and the 322 estimations associated with the seed. The last step is to model the scores evolution according to the acquisition duration for all subjects and for each combination between one seed and one reference. Let us first check assumptions to select a suitable regression model. Assuming the rs-ASL sequence lasts long enough to cover all usage with 24 min 30 s, extrapolation for a duration longer than 24 min 30 s seems superfluous. We are not interested in an explicit formula between scores and acquisition duration, as there is no theoretical model, even in BOLD, on the dependence between acquisition duration and functional network estimation. Moreover, even processed independently, within-subject functional networks estimations have a strong dependency since they come from the same acquisition. Under these three conditions, a local non-parametric regression is very well-suited. We chose to use the LOESS regression model (Cleveland and Devlin, 1988). While the model name is a reference to a geological structure, LOESS is commonly understood as “locally estimated scatterplot smoothing”. LOESS is a local polynomial regression computed on subsets of the whole dataset with each subset being defined for each time-points by a weighted K-nearest neighbors algorithm. We used second degree polynomial functions with a 0.8 span. For a more comprehensive description of LOESS, see Cleveland and Devlin (1988). A LOESS regression curve is computed for each score on each set of 322 overlap scores corresponding to one combination of seed and MSDL references.

## 4. Results

### 4.1. Effect of the Acquisition Duration for the Default Mode Network

**Figure 3:**
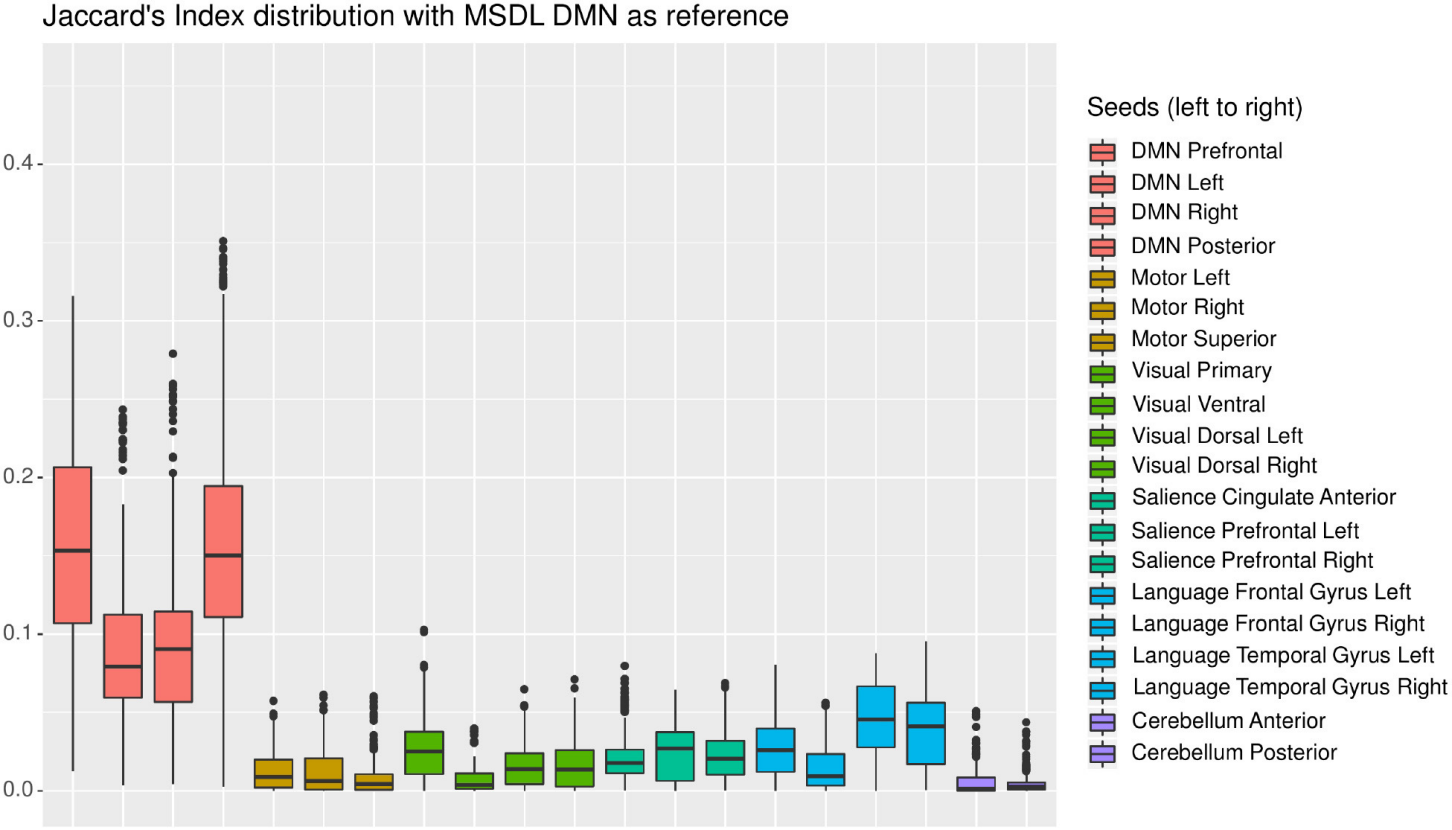
Each boxplot corresponds to one seed and shows the distribution of Jaccard's index between the estimated functional area corresponding to the considered seed on the one hand, and the MSDL DMN reference on the other hand, for all subjects and all durations. The seeds are grouped by color, each corresponding to one of the six functional areas considered. As expected, the seeds located in the expected DMN location (in pink) give the best results.

**Figure 4:**
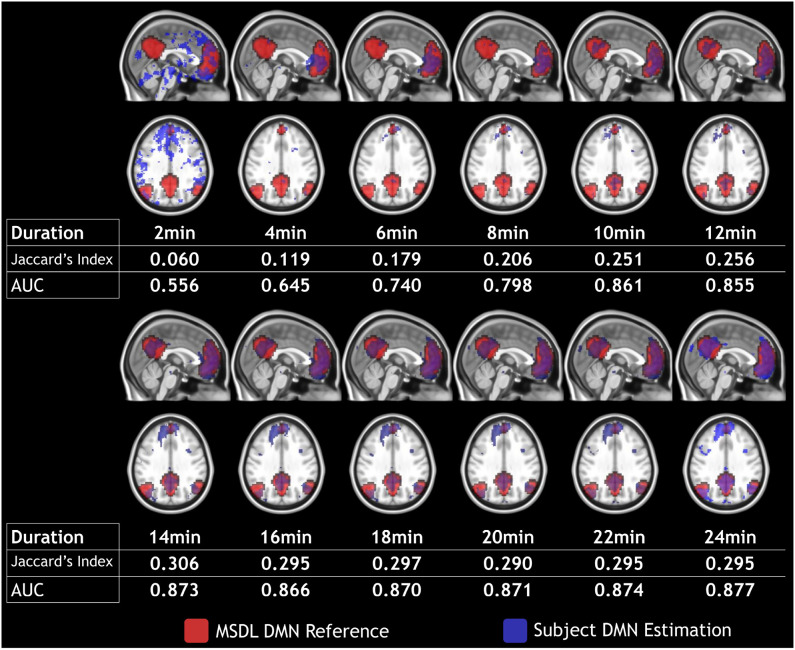
Subject 4 DMN detection (in blue) with prefrontal seed and MSDL DMN reference (in red) over a 2–24 min duration with 2 min steps. Maps are shown in MNI152 space.

**Figure 5:**
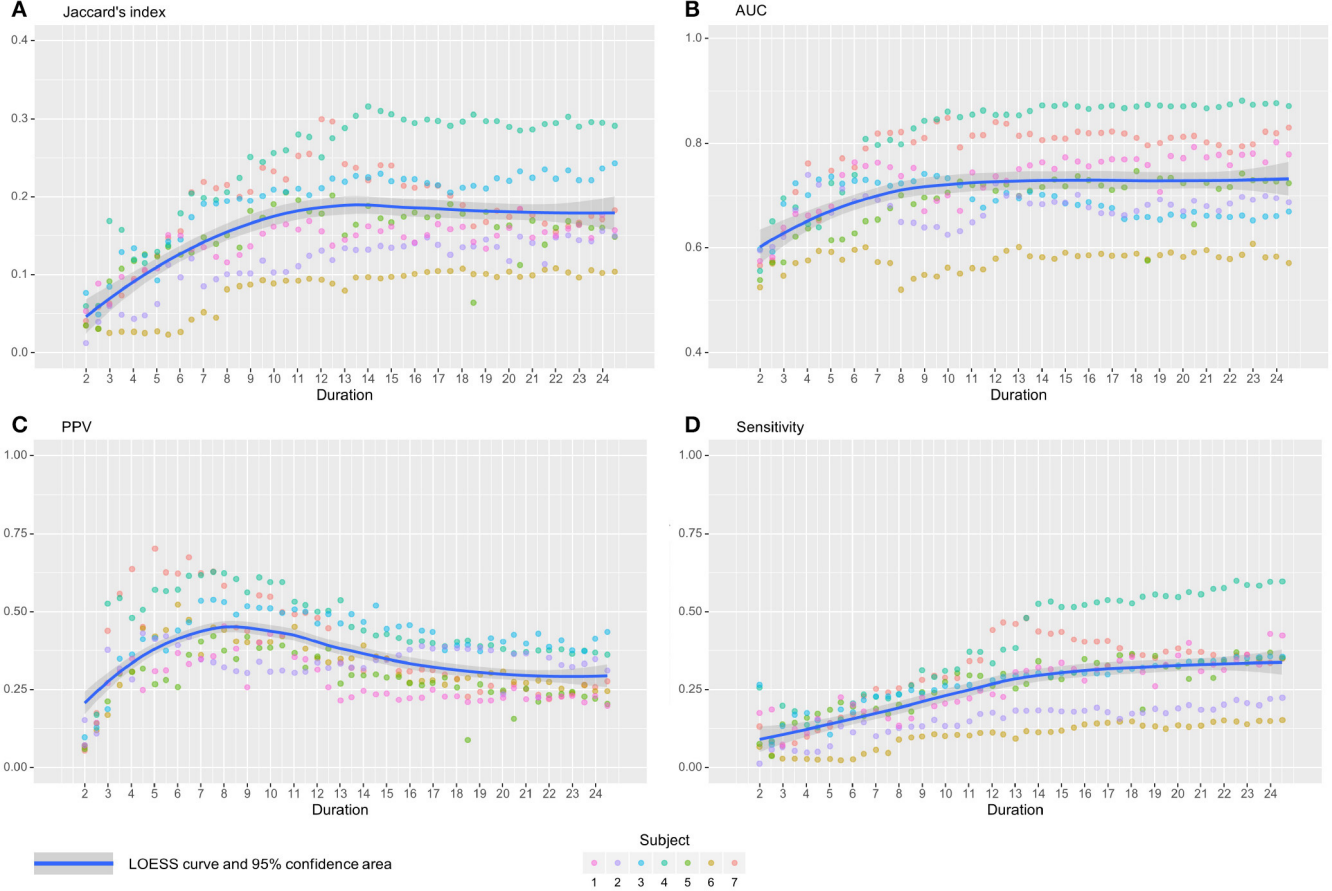
Jaccard's indices **(A)**, AUC **(B)**, Positive Predicted Value **(C)**, and Sensitivity **(D)** evolution with time with their associated LOESS curve for the seed associated with prefrontal DMN. On all subjects, Jaccard's index increases with duration before 10–12 min, then stabilizes. The AUC shows the same trend with an earlier stabilization, around 9–10 min. PPV grows rapidly, reaches a peak at 8 min, then decreases slowly. Finally, Sensitivity increases with time, but more slowly for longer durations. Subjects show different level of response but good correlations.

In this section, we present an in-depth analysis of the DMN. In the set of 20 seeds we used, many should not be inspected when used in combination with MSDL DMN. The main reason is that most of the combinations have no objective basis for detecting the DMN. Otherwise, the seed may have failed to detect precisely the networks it was meant to detect, which is expected with very short acquisition duration. A good way to get an idea of the quality of the overlap between the functional maps associated with a seed and a reference for all durations is to check the boxplots of the Jaccard's index as in Figure 3. Boxplots give an overview of the results for rs-ASL: for the DMN reference, Jaccard's indices have higher values for the seeds placed in order to detect it. Prefrontal and posterior seeds seem to work well, while lateral DMN seeds provide lower scores but still higher than any other seeds. Figure 4 shows the evolution of the estimated DMN with the posterior seed and corresponding scores for one subject. The depiction made by the scores of the overlap between the estimation of the DMN and the MSDL reference matches with the four stages identified in Figure 2. Figure 5 shows the Jaccard's index, AUC, Sensitivity and Predicted Positive Value (PPV) for each subject and at each acquisition duration. We chose to report the positive predicted value rather than specificity for two main reasons. On the one hand, true negatives can have multiple definitions in fMRI since it depends on the voxels considered: the whole volume, only the brain, or any smaller ROI, such as gray matter. Although it is logical to consider only brain voxels for functional activity, this implies an extremely high number of true negatives since the volume of a functional network is 10–100 times smaller than the one of the whole brain. Therefore, the specificity reaches values too high to provide relevant information on similarity between functional areas. On the other hand, like specificity, PPV plays a similar role with respect to sensitivity: specificity gives a complementary information to sensitivity in the totality of voxels whereas PPV gives a complementary information in the union of the reference and the estimated functional area. LOESS on Jaccard's index, as well as on AUC, models quite well what can be observed by looking directly at the functional map as in Figure 2. Jaccard's index seems to stabilize after 12–13 min and AUC at an earlier acquisition duration around 9–10 min. We could have expected sensitivity and PPV to follow the same trend. Actually, sensitivity just grows over time but more slowly for longer durations. Interestingly, PPV reaches a peak in the second stage mentioned above. The seven subjects show different levels of response but good correlations (except for the subject 2 with AUC), i.e., the trend is the same among subject, rather than an average effect induced by the LOESS. Moreover, results observed for the DMN can be generalized for almost every combination of seeds and references as we will see.

### 4.2. Effect of the Acquisition Duration for All Functional Networks

**Figure 6:**
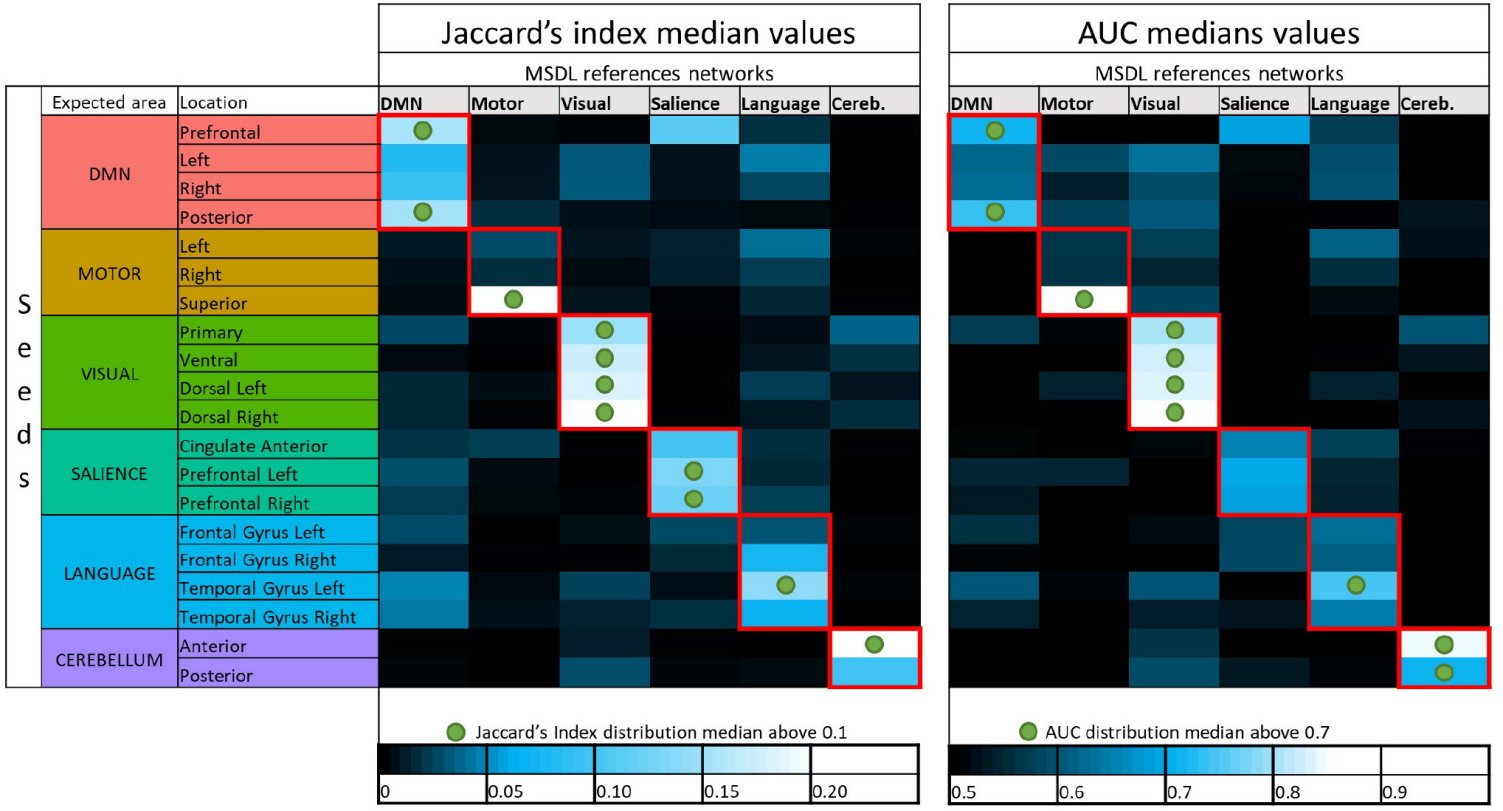
Color maps of median values of Jaccard's indices and AUC for all combinations of seeds and MSDL references. Green circles show where seeds are considered to provide successful detection with respect to our thresholding rules (0.1 for the Jaccard median and 0.7 for the AUC median). The first column of Jaccard's index median values corresponds to the boxplots shown in Figure 3, which showed distribution of Jaccard's index for every seeds in combination with the MSDL DMN reference. The color code used for the seed here is the same as in the boxplots.

As seen for the DMN, many combinations between seeds and references should not be investigated since they are not functionally meaningful and will yield to very low overlapping scores (e.g., prefrontal seed with visual cortex). With more than 6,000 functional maps generated (20 seeds, seven subjects, 46 different acquisition durations), a visual checking of all the maps is not practicable in order to discard seeds that failed to detect corresponding functional networks. In order to investigate only combinations between seeds and references that provide successful detections, we use empirical criteria on the scores distribution. For Jaccard's index, we select the combinations for which at least 50% of observations have *J* ⩾ 0.1. The reasoning behind the choice of the criterion was as follows. With Figure 4 and additional visual inspections on different subjects, we observe that the seed in prefrontal cortex is successful in the detection of the DMN after a certain acquisition duration. Hence, the corresponding boxplot to the prefrontal seeds in Figure 3 provides a Jaccard's index distribution of a successful seed. We assume then that the combinations between seeds and references that have a similar distribution or a better one (i.e., higher values) also correspond to seeds that are able to provide successful estimation of functional networks. We choose to characterize these seeds considered as successful in the estimation, and *a fortiori*, corresponding boxplots, when the median is above 0.1. Using the same considerations on AUC, we also considered the median with a threshold of 0.7. These thresholds on the median values may seem rather low, but let us remember that all the acquisition durations are taken into account, even the shortest ones. Figure 6 shows the median values of the scores for each set of 322 functional networks estimations corresponding to combinations between seeds and references. The two thresholds lead to an almost identical choice for the selection of combinations. All seeds have their best scores with the expected reference, and each of the six functional networks are considered to be sufficiently well-detected with SBA for Jaccard's index in accordance with our selection rules. The AUC suggests as good enough one more seed for cerebellum but considers that salience is not detected well-enough with our set of seeds.

**Figure 7:**
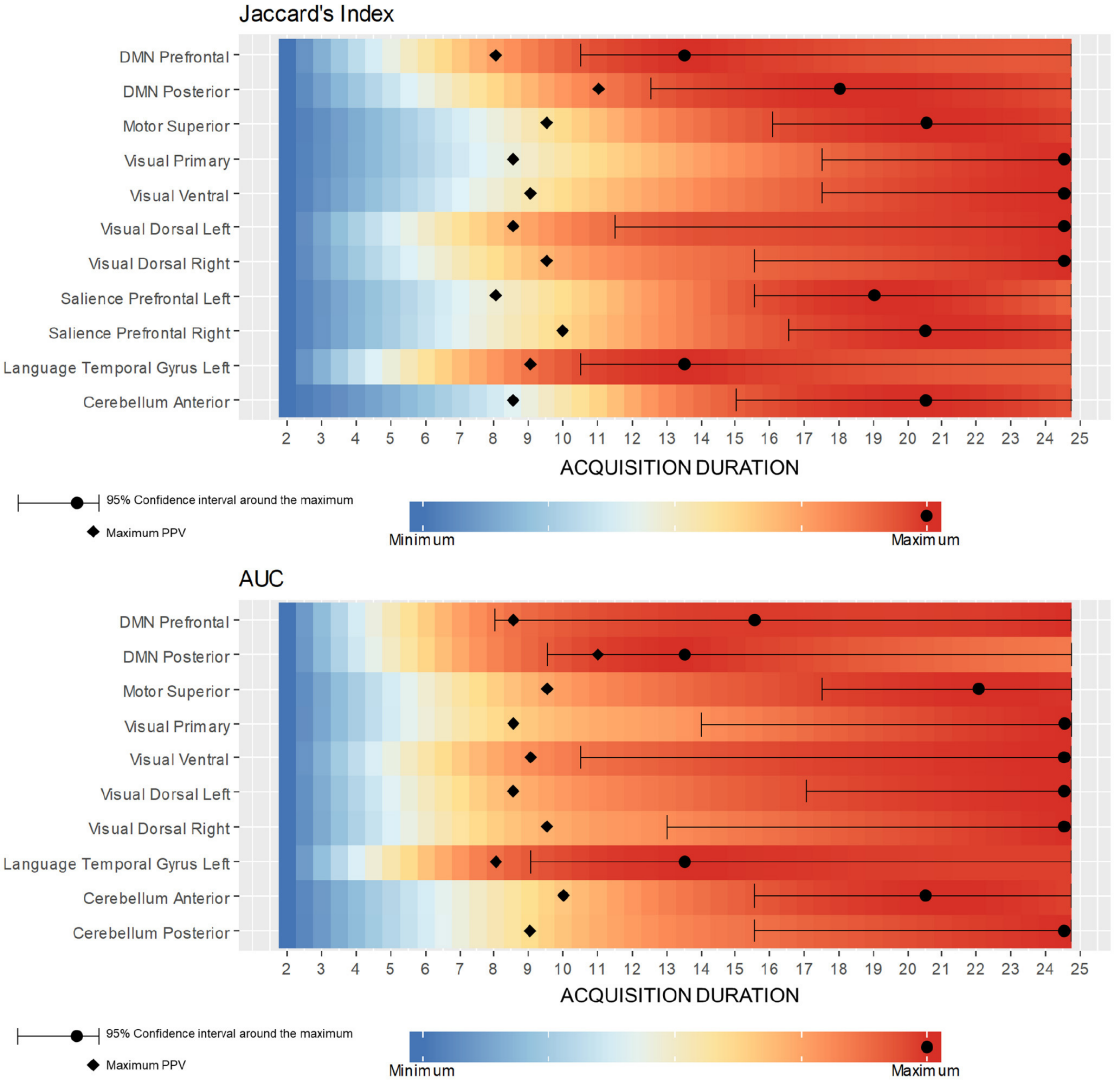
Color maps of Jaccard's Index and AUC LOESS curve value with respect to the acquisition duration for all selected reference/seed combination. Each line corresponds to the computation of one LOESS curve on a subset of 322 functional networks estimations, these subsets of estimations corresponding to a selected seed. Every combination has a rapidly increasing score followed by a stabilization stage. The PPV peak shows on all combinations around 9 min and appears always just before score start stabilizing. Maximum and its 95% confidence interval may be unstable since score variations are often low after PPV peak.

**Figure 8:**
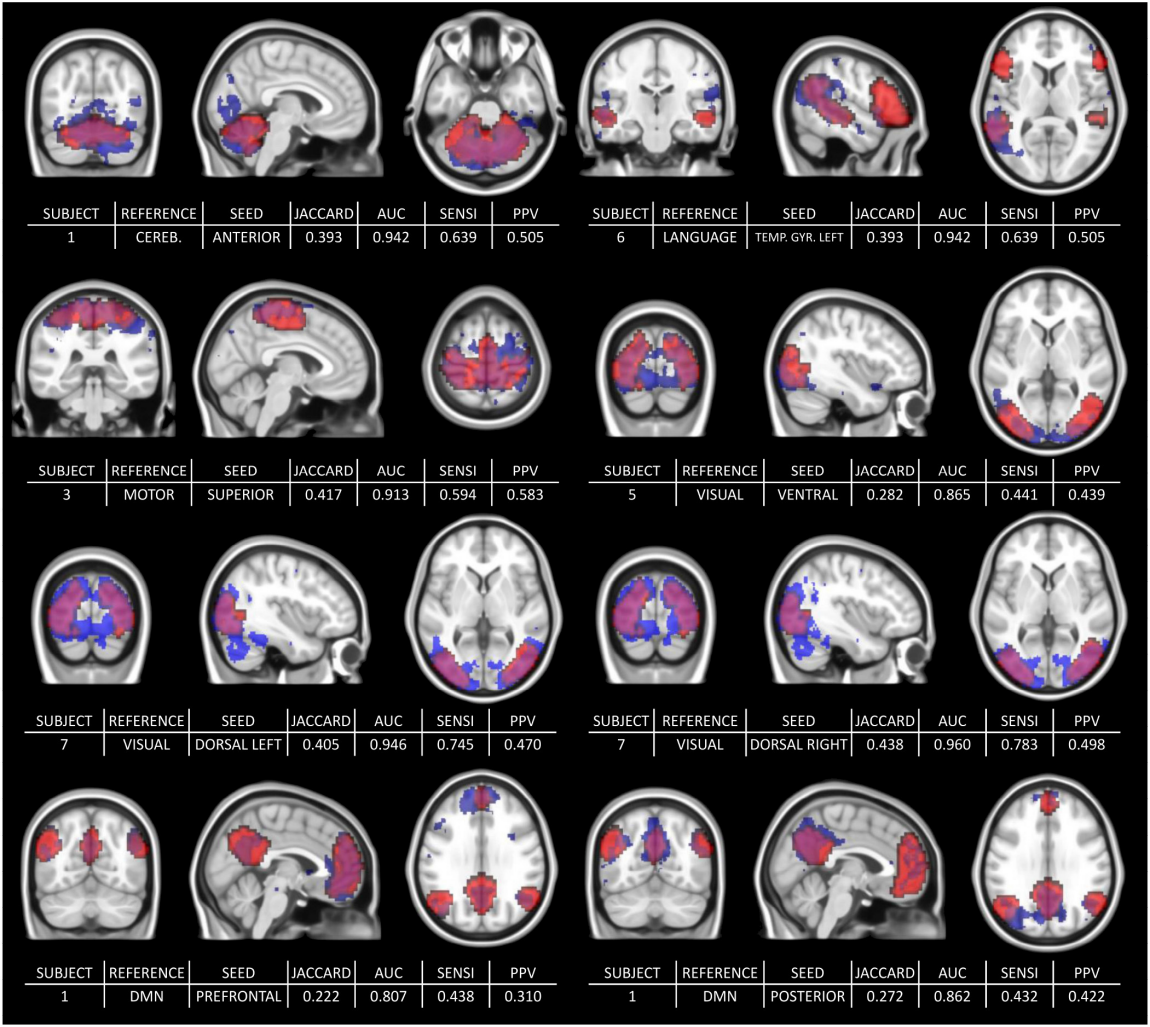
Collection of estimated functional networks at 14 min with the corresponding scores: Jaccard's Index, Area Under the Curve, Sensitivity, and PPV. The two bottom rows show the same subjects and the same reference but with different seeds. The third row shows the estimated (blue) and reference (red) visual network for the same subject (subject 7) but different seeds (“dorsal left” seed on the left and “dorsal right” seed on the right). Similarly, the bottom row shows the same subject (subject 1) with different seeds (“Prefrontal” and “Posterior”).

Figure 7 shows the range of durations where scores are not significantly different from their maximum values (5% risk) for each selected reference/seed combination. Each line corresponds to a LOESS curve computed for one score (Jaccard's index/AUC) and one combination between seed and reference network (322 functional networks estimation per combination). The colors on the heatmap are scaled between minimum and maximum values of the corresponding score and matches with the stages already described for DMN in the previous section. Indeed, for every combination between seeds and references, both scores rapidly increase, and start to stabilize after a certain duration. However, for both measures, the 95% confidence interval around the maximum suggests a later start in the stabilization than suggested directly by the LOESS curve values. While some combinations scores look already stabilized at 12 min, almost all of them are close to their maximum value at 16 min. Figure 8 shows a collection of functional areas obtained at a duration of 14 min. While the language seed struggles to detect spatial components far from the seeds, all the other ones provide good detection of expected functional networks. The two bottom rows show the same subjects and the same reference with different seeds.

## 5. Discussion

Our two objectives were to confirm the feasibility of resting state ASL and to evaluate the influence of the acquisition duration on the estimation of functional areas. Figures 4, 8 with corresponding scores in Figure 6 and in Figure 7 confirm that, even with a classic sequence, and the basic preprocessing and modeling we used, ASL is fully viable as a resting-state method. Regarding the impact of acquisition duration, the most important result is the stabilization of the functional areas estimation after a certain duration for both measures, Jaccard's index and AUC, with a strong inter-subjects correlation (i.e., not a mean-effect induced by the LOESS modeling). Since the acquisition should have the shortest duration possible for clinical implementation, the recommended duration eventually corresponds to the start of the stabilization stage. Stricter definitions of the stabilization stage would lead to longer duration since they would heavily rely on the LOESS curve maximum by considering as stable just a narrow interval around the maximum. However, since after 12–14 min the score variations are low, a slight change in preprocessing or in the population could also lead to an unstable maximum without changing the trend. Relaxed definitions would keep recommended duration stability, but they may consider a functional area as good enough when a human investigator would not. Actually, early stages of acquisition are associated with poor representation of functional areas not in the same connected component that the seed, as we saw with Figures 2, 4. Based on our different results, 14 min seems to be an interesting compromise between shortest duration and best estimation of functional networks.

Regarding the rs-BOLD, others studies have suggested different values for duration/number of volumes: 6 min/72 vol. (Van Dijk et al., 2010), 10 min/250 vol. (Bouix et al., 2017), 12 min/275 vol. (Birn et al., 2013), 25 min/750 vol. (Anderson et al., 2011), and 100 min/2,700 vol. (Laumann et al., 2015). In Termenon et al. (2016), Termenon et al. give as reliable configuration from 14 min/1,200 vol. for 40 subjects to 7 min/580 vol. for 100 subjects. Each paper assesses the effect of acquisition duration in rsBOLD differently: comparison to networks obtained from the longest acquisition (Bouix et al., 2017), connectivity between regions of interest (Birn et al., 2013), reproducibility (Anderson et al., 2011; Laumann et al., 2015), and scoring on graph properties (Termenon et al., 2016). Hence, because of the different modeling, it is hard to say where our rs-ASL suggestion would be placed compared to the different rs-BOLD results, notwithstanding the different sequence and preprocessing. The different quality assessments are not a drawback however, as each work explores different aspects of the impact of the acquisition duration/number of volumes. A natural area of future investigations would be to compare rs-ASL performance to rs-BOLD with the inclusion of acquisition duration/number of volumes as a parameter. Furthermore, as we wanted to also confirm the efficiency of ASL in resting-state fMRI, we naturally focused on a subject scale study and functional networks estimation, but, as rs-BOLD literature shows, numerous aspects of the effect of acquisition duration are yet to be explored.

In order to estimate functional networks, the two most common methods are SBA and Independent Component Analysis (ICA). As ICA is also widely used in rs-fMRI, an investigation of the functional network's spatial stabilization in rs-ASL using the definition of functional connectivity through statistical independence should be beneficial. Indeed, it is not totally guaranteed that statistical independence and linear correlation (or any other alternative modeling of functional connectivity) provide a similar stabilization phenomenon, even if SBA and ICA eventually provide similar functional networks (Cole et al., 2010; Joel et al., 2012).

Note that the DMN, the sensori-motor cortex, and the cerebellum have an almost consensual spatial definition among the authors, unlike language, visual and salience, which show a greater spatial variability (see for example http://neurosynth.org/). As we provide an evaluation only with one set of references (from the MSDL), this could be a limitation of our work and could call into question the score values we obtained. However, the spatial variability of the areas of interest in atlases is low enough to change only the scores but not the trend observed in this paper. Indeed, overlap scores can be interpreted as distances, and, in that sense, we measured the distance between functional networks estimation and fixed points (the set of references). Changing the set of references modifies scores values but not the stabilization phenomenon, as the references are still fixed. Score values were used for seeds validation by thresholding overlap scores with MSDL references. After considering only the successful seeds, the score values matter less than their stabilization after a certain acquisition duration. Regarding the score values, using a ground truth built from subjects would most likely bias the scores toward higher values but could also question the validity of such ground truth, where confidence in atlases is much stronger. Niazy et al. (2011), Zhu et al. (2013) report that a Sørensen-Dice score of 0.3 corresponds to a good overlap, regarding the reproducibility of functional networks between rs-ASL and rs-BOLD (i.e., with ground-truth built from same set of subjects). As a Sørensen-Dice score of 0.3 corresponds to a Jaccard's index of 0.176^1^, and our subjects are independent from the MSDL BOLD atlas, the median values of Jaccard's indices we obtained in Figures 3, 6, or the values we obtained in Figures 4, 8, confirm *a posteriori* the criteria for seeds selection, and *a fortiori*, rs-ASL's ability to provide good estimation of functional networks.

Preprocessing influence should also be considered as positive: since we use typical and basic preprocessing, more advanced techniques should foster functional networks estimation, and therefore, provide the same or an earlier stabilization, still keeping our suggestion as a sufficient duration. The same is true for ASL readout approach. Using a 3D readout is probably an improvement in our sequence, as it tends to have higher SNR compared to 2D EPI, but not every investigator has access to 3D sequences (Alsop et al., 2015). Not to mention readout approach, ASL is very sensitive to changes in its parameters. Since we are studying the influence of acquisition duration for a given set of parameters, the suggested sufficient duration of 14 min for rs-ASL could be strongly influenced by the sequence parameters. Although the influence of each of them is to be kept in mind, most of them should not disturb the investigators, as most of them have a specific bibliography that goes well-beyond the issue of the acquisition duration. However, two sequence parameters may have a deep impact on our results: post-labeling delay (PLD) and repetition time (TR). For the PLD, the 1,250 ms duration we used for the first slice corresponds to 1712.5 ms at the median slice. Shorter PLD, like 600 ms, seems to give a better functional estimation (Viviani et al., 2011; Liang et al., 2012, 2014). We kept the PLD quite long, close to the 1,800 ms at the median slice, which is recommended for best estimation of CBF (Alsop et al., 2015; Chen et al., 2015). Indeed, the main advantage of ASL is ultimately to compute CBF, although we focused on functional areas estimation in this paper. The critical parameter in our opinion is repetition time. It defines the sample frequency of the resting-state signal and turns our 14 min suggestion into 240 volumes since we only work numerically on the signal. Its variation may shift the stabilization step toward a higher/lower number of volumes and hence a longest/shortest duration, without changing the stabilization of the functional networks estimation after a certain number of volumes (i.e., same signal but different sampling frequency). Moreover in rs-ASL, TR values are typically between 3 and 5 s, which is too wide to assume the locally linear dependence between TR and optimal duration. As a pioneer work on the effect of acquisition duration in rs-ASL, we focused more on the modeling rather than investigating the influence of the TR. In rsBOLD, the TR has already been shown to be able to impact estimation considering a fixed number of volumes (Wu et al., 2011), but for rs-ASL a specific investigation of TR in combination with the number of volumes would be highly valuable.

## 6. Conclusion

Usual sequence, preprocessing, and SBA managed to reconstruct the six typical functional networks of interest at the subject scale, which confirms the feasibility of ASL as a rsfMRI technique. The main objective was to find the amount of data to acquire in rs-ASL to properly estimate functional networks, considering that clinical implementation requires the shortest possible duration. Our results show that functional networks estimations stabilize after a certain number of volumes/duration. For our set of sequence parameters, we suggest 240 volumes/14 min to achieve an overall stabilization. Any method that improves the detection of functional networks is likely to provide an earlier stabilization start, i.e., a lower number for volume/duration. Hence, since we use a basic and typical sequence, preprocessing and estimation, 240 volumes/14 min should be enough for most rs-ASL usage. Last, the exploration of the impact of the TR and PLD in combination with acquisition duration was beyond the scope of this article but would be highly beneficial for sequence implementation since we forecast them to be the two parameters that may shift the stabilization start toward higher number of volumes.

## Data Availability Statement

In accordance with the consent form signed by the subjects, authors are not allowed to share MRI acquisitions. However, requests to see the raw data can be sent to the corresponding author. For the preprocessing steps we used Matlab CONN toolbox (www.nitrc.org/projects/conn, RRID:SCR_009550) (Whitfield-Gabrieli and Nieto-Castanon, 2012). The rest of the code used for evaluation scores and LOESS regression is available upon request from the corresponding author.

## Ethics Statement

The studies involving human participants were reviewed and approved by Ethics Committee of the University Hospital of Rennes CHU Rennes Hôtel-Dieu, 2 rue de l'Hôtel-Dieu, CS 26419, 35064 Rennes Cedex, France. The patients/participants provided their written informed consent to participate in this study.

## Author Contributions

CV designed the MR acquisition protocol with the help of the Neurinfo MRI research facility, designed the research, performed the research, analyzed the data, and wrote the paper. PM, IC, and CB supervised all the steps and corrected the paper. All authors contributed to the article and approved the submitted version.

## Conflict of Interest

The authors declare that the research was conducted in the absence of any commercial or financial relationships that could be construed as a potential conflict of interest.

## Footnotes

^1^Jaccard = Dice/(2 − Dice).

## Appendix—Seed Location in MNI152

**Table:**

**Expected networks**	**Seed**	**Location in MNI152**
DMN	Prefrontal	(1, 55, −3)
DMN	Left	(−39, −77, 33)
DMN	Right	(47, −67, 29)
DMN	Posterior	(1, −61, 38)
Motor	Left	(−55, −12, 29)
Motor	Right	(56, −10, 29)
Motor	Superior	(0, −31, 67)
Visual	Primary	(2, −79, 12)
Visual	Ventral	(0, −93, −4)
Visual	Dorsal left	(−37, −79, 10)
Visual	Dorsal right	(38, −72, 13)
Salience	Cingulate anterior	(0, 22, 35)
Salience	Prefrontal left	(−32, 45, 27)
Salience	Prefrontal right	(32, 46, 27)
Language	Frontal gyrus left	(−51, 26, 2)
Language	Frontal gyrus right	(54, 28, 1)
Language	Temporal gyrus left	(−57, −47, 15)
Language	Temporal gyrus right	(59, −42, 13)
Cerebellum	Anterior	(0, −63, −30)
Cerebellum	Posterior	(0, −79, −32)
